# Characteristics of human oral microbiome and its non-invasive diagnostic value in chronic kidney disease

**DOI:** 10.1042/BSR20210694

**Published:** 2022-05-10

**Authors:** Shiyuan Guo, Ge Wu, Wenli Liu, Yajuan Fan, Wengang Song, Jian Wu, Dan Gao, Xi Gu, Sanhui Jing, Quanquan Shen, Lingyan Ren, Yindi Dong, Zhangsuo Liu

**Affiliations:** 1 Department of Nephrology, the First Affiliated Hospital of Zhengzhou University, Zhengzhou 450052, China; 2 Clinical Laboratory Diagnostics, Medical Technology College, Beihua University, Jilin 132013, China; 3 Department of Nephrology, the Central Hospital of Zhumadian, Zhumadian, Henan 463000, China; 4 College of Public Health, Zhengzhou University, Zhengzhou 450052, China; 5 Department of Nephrology, Heze Chinese Medicine Hospital, Heze 274000, China; 6 Department of Nephrology, Zhejiang Provincial People's Hospital, People's Hospital of Hangzhou Medical College, Hangzhou, Zhejiang 310014, China; 7 Department of Nephrology, the First Affiliated Hospital of Huzhou Teachers College, the First People's Hospital of Huzhou, Huzhou, Zhejiang 313000, China

**Keywords:** Chronic kidney disease, Microbial biomarker, Miseq sequencing, Non-invasive diagnostic value, Oral microbiome

## Abstract

**Background:** Morbidity of chronic kidney disease (CKD) is increased, with many complications and high mortality rates. The characteristics of oral microbiome in CKD patients have not been reported. This study aims to analyze the oral microbiome, and to demonstrate the potential of microbiome as noninvasive biomarkers for CKD patients.

**Methods:** The study collected 253 oral samples from different regions of China (Central China and East China) prospectively and finally 235 samples completed Miseq sequencing, including 103 samples from CKD patients and 132 healthy controls (HCs).

**Results:** Compared with HCs (*n*=88), the oral microbial diversity in CKD patients (*n*=44) was increased. Fourteen genera including Streptococcus, Actinomyces and Leptotrichia were enriched, while six genera including Prevotella and Haemophilus were decreased in CKD patients. Moreover, 49 predicted microbial gene functions including arginine metabolism and tryptophan metabolism increased, while 55 functions including Ribosome and DNA repair recombination proteins decreased. Furthermore, correlation analysis demonstrated that 38 operational taxonomic units (OTUs) were closely related to 5 clinical indicators of CKD. Notably, 7 optimal biomarkers were identified using random forest model, and the classifier model respectively reached an area under the curve (AUC) of 0.9917 and 0.8026 in the discovery and validation phase, achieving a cross-region validation.

**Conclusions:** We first illustrated the characteristics of the oral microbiome of patients with CKD, identified the potential of oral microbial makers as noninvasive tools for the diagnosis of CKD and achieved cross-region validation.

## Introduction

Chronic kidney disease (CKD), with increased morbidity, high mortality rates and many complications, would eventually leads to end-stage renal disease (ESRD) [1]. Furthermore, CKD is not only one of the major diseases that threatens public health but also seriously affects patient quality of life [2,3]. Meanwhile, the progression of CKD is insidious. The only treatments for patients with ESRD are hemodialysis, peritoneal dialysis, and kidney transplantation, which would bring a serious economic burden to the patient’s family and society. Therefore, it is crucial to discover new diagnosis and treatment methods for CKD.

The oral microbiome is one of the most diverse microbial communities in the human body [4]. It is composed of greater than 700 bacteria or system types [4], and divided into 4 floras including dorsal tongue flora, salivary flora, dental plaque flora and subgingival plaque flora. The composition and characteristics of oral microbiome were different according to the different locations [5–7], and the composition of tongue coat flora is more stable and contains components from other parts [8,9]. Adults produce >1000 ml of saliva per day, almost all of which enters the gastrointestinal tract. Thus, salivary bacteria have great opportunity to enter and colonize the intestine, suggesting they can participant in the development of the intestinal microbial community structure to a certain extent [10]. Current studies have proved that the intestinal flora of CKD patients has changed, so we speculate that there may be some differences in the oral microbiome of CKD patients versus healthy people.

The diagnostic potential of the oral microbiome for rheumatoid arthritis (RA) [11], liver cancer [12] and pancreatic head cancer [13] has been confirmed. However, its diagnostic potential for CKD needs further research. In this study, we prospectively collected 253 oral samples from different regions of China, of which 235 samples were completed to Miseq sequencing. In the discovery phase, we characterized the oral microbiome, identified microbial markers, and constructed CKD classifier in the 88 healthy controls (HCs) and 44 CKD. Then, we verified the diagnostic efficacy of the CKD classifier in the validation phase.

## Materials and methods

### Enrolled patients

All enrolled patients met the diagnostic criteria for CKD and had not undergone renal replacement therapy. Diagnosis and staging were carried out in accordance with the recommendations of the Kidney Disease Outcomes Quality Initiative (K/DOQI) of the National Kidney Foundation [14]. Criteria for CKD are shown in the table.

**Table:** Criteria for chronic kidney disease

**Markers of kidney damage (>1 for >3 months)**
Albuminuria (AER 30 mg/d; ACR 30 mg/g)
Urinary sediment abnormalities
Electrolyte and other abnormalities due to tubular disorders
Abnormalities detected by histology
Structural abnormalities detected by imaging
History of kidney transplantation
**Decreased GFR (for >3 months)**
GFR 60 ml/min per 1.73 m^2^ (GFR categories G3a–G5)

Abbreviations; ACR, albumin– creatinine ratio; AER, albumin excretion rate; GFR, glomerular filtration rate.

Further, the following criteria were met: (a) no consumption of antibiotics and/or probiotic preparations in the past month; (b) no previous history of liver diseases, diabetes and tumors; (c) no history of oral ulcers in the past month; (d) no CKD-related drug therapy has been initiated, such as immunosuppressant, glucocorticoid and statins; (e) no abuse of alcohol/drug/tobacco.

The health controls (HCs) group consisted of 132 healthy volunteers who visited our hospital for their annual physical examination. They had to meet the following criteria to be included in the experiment: (a) hemoglobin, liver function, kidney function, electrolytes, urine, stool and serological tests (including detection of HBsAG, and antibodies against hepatitis C virus, Treponema pallidum and human immunodeficiency virus) were normal; (b) no diabetes, obesity, cardiovascular diseases, tumor, oral disease and digestive system disease; (c) did not used antibiotics and/or probiotics 8 weeks before the sample was collected; (d) no abuse of alcohol/drug/tobacco.

HC were selected whose age, gender, and BMI index matched those of CKD patients enrolled in the study. All clinical indicators, lifestyle, drug use and past medical records that need to be observed in the trial come from the hospital information system and questionnaires.

### Sample collection

After successful screening, we immediately collected samples from the participants. Before sampling, the enrollee was asked to rinse and gargle twice with sterile water. Oral samples were collected by professional stemmatologist using pharyngeal swabs, which collect the oral samples from the posterior middle area to the anterior middle area, then immersed in phosphate buffered solution. The throat swab sponge was squeezed on the tube wall several times for 20 s to ensure the transfer of microorganisms from the swab to the buffer. The samples were quickly transferred to the laboratory for centrifugation, and the supernatant was discarded. The pellets were stored at −80°C within 1 h.

### DNA extraction, 16S rRNA polymerase chain reaction (PCR) amplification and sequencing

The Qiagen Mini Kit (Qiagen, Hilden, Germany) was used to extract microbial DNA from oral samples, according to the manufacturer’s instructions [15–17]. Quantitative DNA analysis was performed using the Qubit 2.0 Fluorometer (Invitrogen, Carlsbad, CA, U.S.A.), and agarose gel electrophoresis was employed to estimate molecular size. Finally, the DNA sample of oral microbiome was diluted to 10 ng/μl for further analysis.

The universal target V3 to V4 region of the 16S rRNA gene was amplified by PCR using the primer 341F/805R, which used the extracted DNA as a template. The 4 PCR reactions will follow the following cycling conditions: 94°C for 4 min, followed by 35 cycles of 94°C for 30 s, 55°C for 30 s and 72°C for 1 min, followed by a final extension at 72°C for 10 min [13]. The PCR products were then mixed with l0 uL PCR amplification solution and subjected to 2% agarose gel electrophoresis at a voltage of 100 V. After electrophoresis for 30–60 min, the amplification results were visualized under UV. The bands were extracted by AxyPrepDNA gel (Axygen, CA, U.S.A.), and the bands were purified by MinElute kit (QIAGEN). The DNA product obtained after purification was quantified using a fluorescence assay kit (Quant-iT PicoGreen, Invitrogen). The amplicons were sequenced using the Illumina MiSeq sequencing platform (Shanghai Mobio Biomedical Technology Co. Ltd., China). The raw reads were deposited into the European Nucleotide Archive database (Study accession Number: PRJNA557511 (HCs) and PRJNA649074 (CKD)).

The FLASH v1.2.10 software was used to read the paired ends of the original DNA fragments and merge them into a single sequence. After strict screening, they are classified according to their specific barcodes [12]. The UPARSE software (version 7.1 http://drive5.com/uparse/) was used to remove chimera and classified Operational taxonomic units (OTUs) based on 97% similarity.

### Bioinformatics analysis

Clean data was extracted from Raw data using USEARCH (version 7.0.1090). Alpha diversity metrics (ACE estimator, Chao 1 estimator, Shannon–Wiener diversity index and Simpson diversity index) were assessed by using Mothur v1.42.1. Principle co-ordinates analysis (PCoA), principal component analysis (PCA) and nonmetric multidimensional scaling (NMDS) were used to analyze microbial community changes between the CKD and HC groups, which were conducted by the R (version 3.6.0) package (http://www.R-project.org/). Heatmap was accomplished by the heatmap builder to distinguish the key variables. A heatmap that identified key variables was accomplished by the heatmap builder. Linear disciminant analysis (LDA) effect size (LEFse) (http://huttenhower.sph.harvard.edu/lefse/) was used to identify and rank statistically different biomarkers in the two groups, which was depicted in graph form. And LDA was used to evaluate the influence of species with significant differences [LDA score (log10) = 2 as cut-off value]. Phylogenetic Investigation of Communities by Reconstruction of Unobserved States (PICRUSt) (http://picrust.github.io/picrust/tutorials/algorithm_description.html) was employed to predict the metabolic function profile of the oral microbiome from the 16S rRNA gene sequences. A 5-fold cross-validation analysis has been performed using rfcv function in R-package ‘randomForest’ (R version 3.2.1) to valid the key discriminatory OTUs which selected by random forest analysis and sift through the minimum OTU combination. The combination with the lowest error rate and the smallest number is selected as the optimal OTUs, which is used to calculate the probability of disease (POD) index of the discovery phase and the validation phase. Finally, the pROC software package was used to construct Receiver operating characteristic (ROC) curves to evaluate the quality of the classification models.

### Statistical and bioinformatics analysis

Statistical analysis was performed using the SPSS V.21.0 for Windows (SPSS, Chicago, Illinois, U.S.A.). Continuous variables are expressed as mean (standard deviation) or median (interquartile range). Categorical variables are expressed as percentages. Differences between subjects in CKD and HCs were compared by using Student’s *t*-test for normal continuous variables, Wilcoxon rank-sum test for non-normal continuous variables, and Chi-square test or Fisher’s exact test for categorical variables. And the statistically significance was defined as *P*/*Q*<0.05 (two-tailed), without post-analysis and α adjustment.

## Results

**Figure 1:**
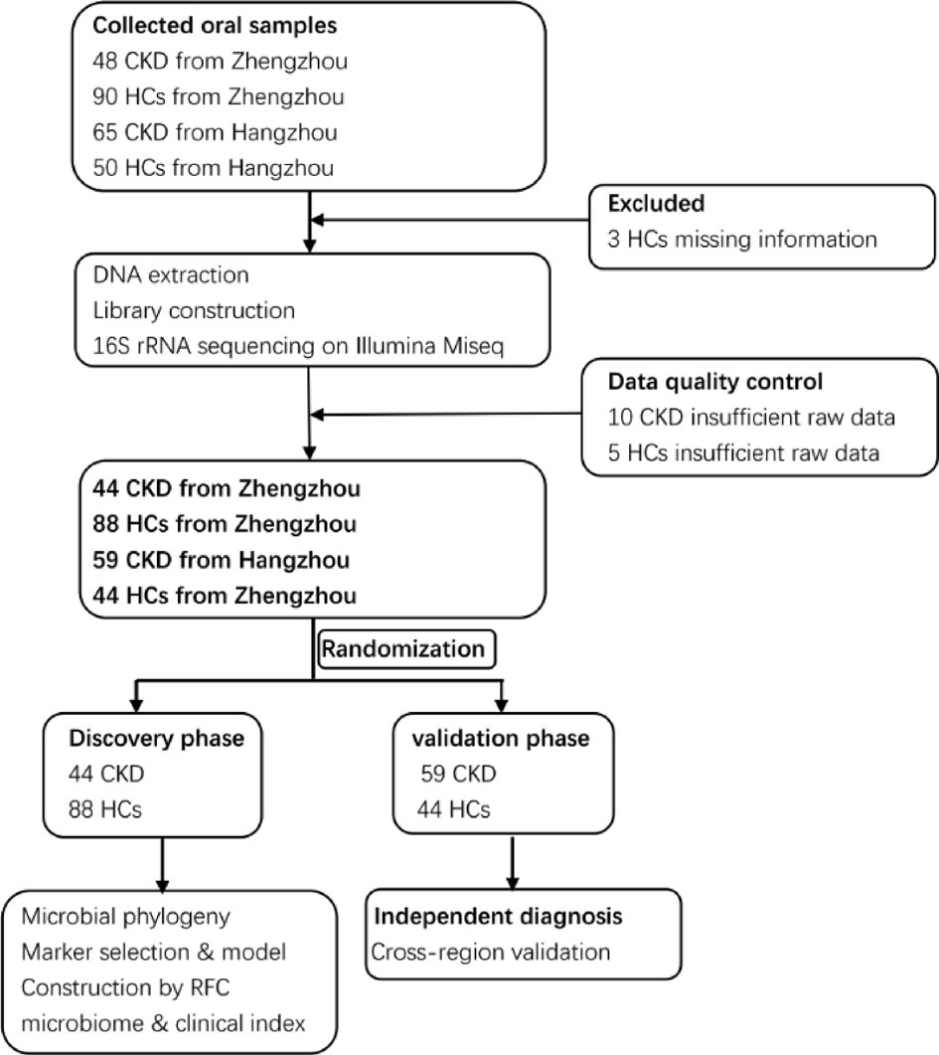
Study design and flow diagram A total of 253 oral samples from different parts of China were collected prospectively. After a rigorous diagnosis and exclusion procedures, 235 samples completed the Miseq sequencing, 44 patients with CKD and 88 healthy controls samples from Zhengzhou, China, and 59 patients with CKD and 44 HCs samples from Hangzhou, China, were included. In the discovery phase, we characterized oral microbiome between 44 CKD and 88 HCs and identified the microbial markers and constructed a CKD classifier by a random forest classifier model between CKD and HCs. In the validation phase, 59 CKD and 44 HCs were used to validate diagnosis efficacy of CKD classifier and as independent diagnostic cohort to validate the diagnostic efficiency of CKD classifier; CKD, chronic kidney disease; HC, healthy control; RFC, random forest classifier model.

A total of 253 oral samples from different parts of China were collected prospectively. After a rigorous diagnosis and exclusion procedures, 235 samples completed the Miseq sequencing, 44 patients with CKD and 88 healthy controls samples from Zhengzhou, China, and 59 patients with CKD and 44 HCs samples from Hangzhou, China, were included (Figure 1). In the discovery phase, we described the microbiome characteristics of the CKD group (*n*=44) and HCs group (*n*=88) oral samples, and found key microbial markers, and successfully constructed a CKD diagnostic classifier. In the validation phase, we validated the diagnostic potential of the constructed classifier for disease by oral samples of CKD groups (*n*=59) and HCs groups (*n*=44), and achieved cross-regional validation.

### Clinical characteristics of the participants

Compared with the HCs, blood urea nitrogen (BUN), serum creatinine (Scr) and uric acid (UA) levels were significantly increased (*P*<0.001), and estimated glomerular filtration rate (eGFR) was significantly decreased (*P*<0.001) in the CKD group. In addition, albumin (ALB) levels were significantly reduced in the CKD group (*P*<0.001), while their triglyceride serum levels were significantly increased (*P* = 0.05 and *P* = 0.001, respectively) (Table 1). Detailed clinical data of all subjects are listed in the Supplementary Data S1.

**Table 1:** Clinical characteristics of the enrolled participants

Clinical indexes	Discovery (*n*=132)		P (Control vs. CKD)	Validation (*n*=103)		P (Control vs. CKD)
	Control (*n*=88)	CKD (*n*=44)		Control (*n*=44)	CKD (*n*=59)	
Age (year)	46.57 ± 7.88	41.05 ± 16.36	0.125	60.20 ± 8.13	60.85 ± 13.98	0.786
Gender						
Female	39(44.32%)	17(38.64%)	0.543	22(50.00%)	34(57.63%)	0.442
Male	49(55.68%)	27(61.36%)	–	22(50.00%)	25(42.37%)	–
BMI (kg/m^2^)	23.81 ± 2.11	23.92 ± 4.21	0.853	23.54 ± 2.39	24.18 ± 3.33	0.216
Hypertension						
Yes	0(0.00%)	26(59.09%)	–	22(50.00%)	30(66.00%)	–
No	88(100.00%)	40(90.91%)	–	22(50.00%)	29(34.00%)	–
WBC (×10^9^/L)	5.64 ± 1.24	6.38 ± 1.20	0.001	5.28 ± 0.93	3.95 ± 0.76	<0.001
RBC (×10^12^/L)	4.65 ± 0.44	4.18 ± 0.79	0.001	4.57 ± 0.40	6.72 ± 2.86	<0.001
24 h UTP (g)	ND	3.24 ± 2.548	–	ND	1.55 ± 2.45	–
Albumin (g/L)	48.00 ± 2.61	37.58 ± 7.98	<0.001	48.10 ± 2.42	40.24 ± 10.11	<0.001
Urea nitrogen (mmol/l)	4.93 ± 1.18	11.01 ± 9.98	<0.001	4.48 ± 0.73	ND	–
Scr (umol/l)	68.85 ± 14.16	205.16 ± 224.78	<0.001	65.36 ± 12.87	147.02 ± 137.32	<0.001
UA (umol/l)	280.68 ± 73.76	395.09 ± 128.07	<0.001	266.66 ± 68.48	399.36 ± 137.10	<0.001
eGFR (ml/min/1.73m^2^)	102.69 ± 11.00	67.32 ± 42.41	<0.001	105.58 ± 8.58	60.39 ± 36.20	<0.001
TG (mmol/l)	1.20 ± 0.43	2.57 ± 2.61	0.001	1.17 ± 0.43	1.92 ± 0.96	<0.001
Calcium (mmol/l)	ND	2.22 ± 0.18	–	ND	2.29 ± 0.21	–
Phosphate (mmol/l)	ND	1.37 ± 0.43	–	ND	1.21 ± 0.33	–
CKD study stage						
1-2 stage	ND	23(52.27%)	–	ND	28(47.46%)	–
3-4 stage	ND	13(29.55%)	–	ND	27(45.76%)	–
5 stage	ND	8(18.18%)	–	ND	4(6.78%)	–

Categorical variables are expressed as group percentages and were compared among samples using Pearson’s χ2 or Fisher’s exact test. Continuous data are presented as either the mean ± standard deviation (SD) for normally distributed variables or the median (inter quartile range) for non-normally distributed variables. Independent sample analysis of variance was used for comparisons of parametric data, whereas the Mann–Whitney *U* test was used for comparisons of nonparametric data. All of these statistical tests were two-sided, with a significance level of <0.05. Statistical analyses were conducted using SPSS version 21.0 for Windows (SPSS Inc., Chicago, IL). Abbreviations: 24 UTP, 24 h urine protein quantitation; CKD, chronic kidney diseases; eGFR, estimated glomerular filtration rate; LDL, low density lipoprotein; ND, no detection; RBC, red blood cell; Scr, serum creatinine; T-chol, total cholesterol; TG, triglyceride; UA, uric acid; WBC, white blood cell.

### Increased microbial diversity in the oral of CKD patients

The rarefaction curves indicated that the microbial richness of the sampled oral was near saturation at the applied sequencing depth (Supplementary Figure S1a), which was sufficient to identify most of the bacterial community members of each individual microbiome. The Shannon–Weiner curve based on OTUs had already been flat, indicating that our sequencing depth had already been adequate (Supplementary Figure S1b). The rank-abundance curve demonstrated the microbial richness was higher in the CKD group than the HCs group (Supplementary Figure S1c).

**Figure 2:**
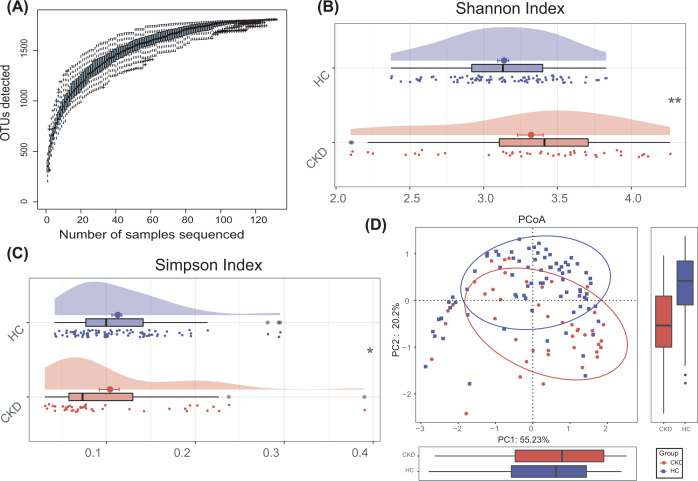
Oral microbial diversity of patients with CKD was increased in the discovery phase (**A**) The Specaccum species accumulation curses (Figure 2A) indicated that OUT richness approached saturation in all samples, and shown adequate sampling. (**B,C**) The cloudplots show that microbiomes diversity differences according to the Shannon index and Simpson index between the CKD (*n*=44) and HCs (*n*=88). Plot parameters, the ‘bold dot’ symbol represents median value, the upper and lower ranges of the scatter in the figure represent 25% and 75%, respectively. (**D**) PCoA for diversity clustering analysis of CKD (*n*=44) and HCs (*n*=88) oral microorganisms. Each symbol represents a sample (red, CKD; blue, HC). *P<0.05; **P<0.01 and ***P<0.001; CKD, chronic kidney disease; HC, healthy control; PCoA, Principle co-ordinates analysis.

The Specaccum species accumulation curses (Figure 2A) revealed that OUT richness approached saturation in all samples. As estimated by the Shannon index (Figure 2B) and the Simpson index (Figure 2C), the oral microbial diversity was significantly increased in CKD compared with healthy controls (*P*<0.01, *P*=0.01, respectively, Supplementary Data S2). No significant differences in community richness (estimated by Chao and ace indices) was observed between CKD and HCs (Supplementary Figure S2). Additionally, the principal coordinate analysis (PCoA) based on distribution of the OTUs was conducted to illustrate the microbiome space of different samples. The oral microbiome composition was significantly different between CKD and HCs (Figure 2D).

### Alterations of oral microbiome in CKD patients

**Figure 3:**
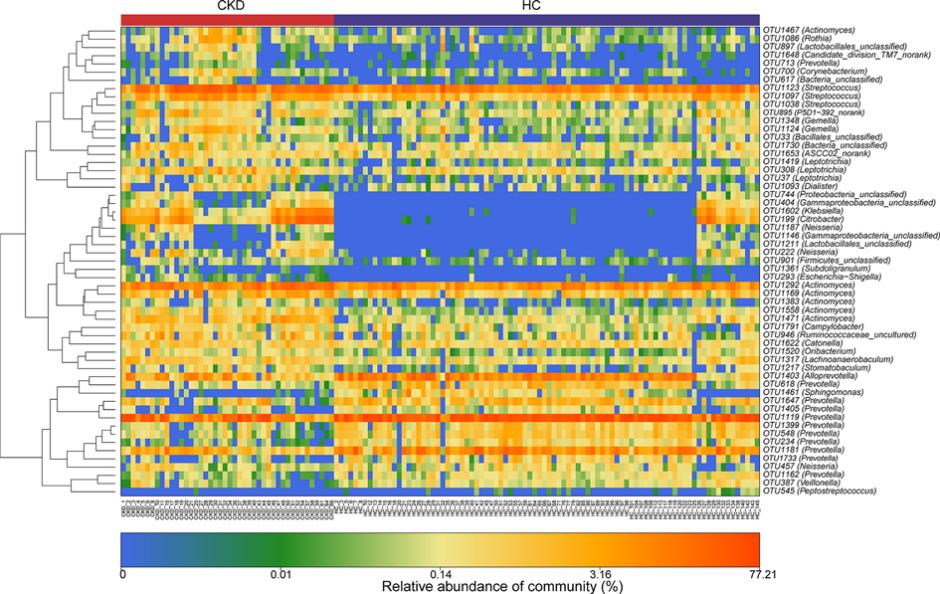
Heatmaps for relative abundances of differential OTUs between CKD (*n*=44) and HCs (*n*=88) For each sample, each row shows the relative abundance data for the discriminating OTU listed on the right side of the figure. The relative abundance of each OTU was used to plot the heatmap. (blue, low abundance; red, high abundance); CKD, chronic kidney disease; HC, healthy control.

**Figure 4:**
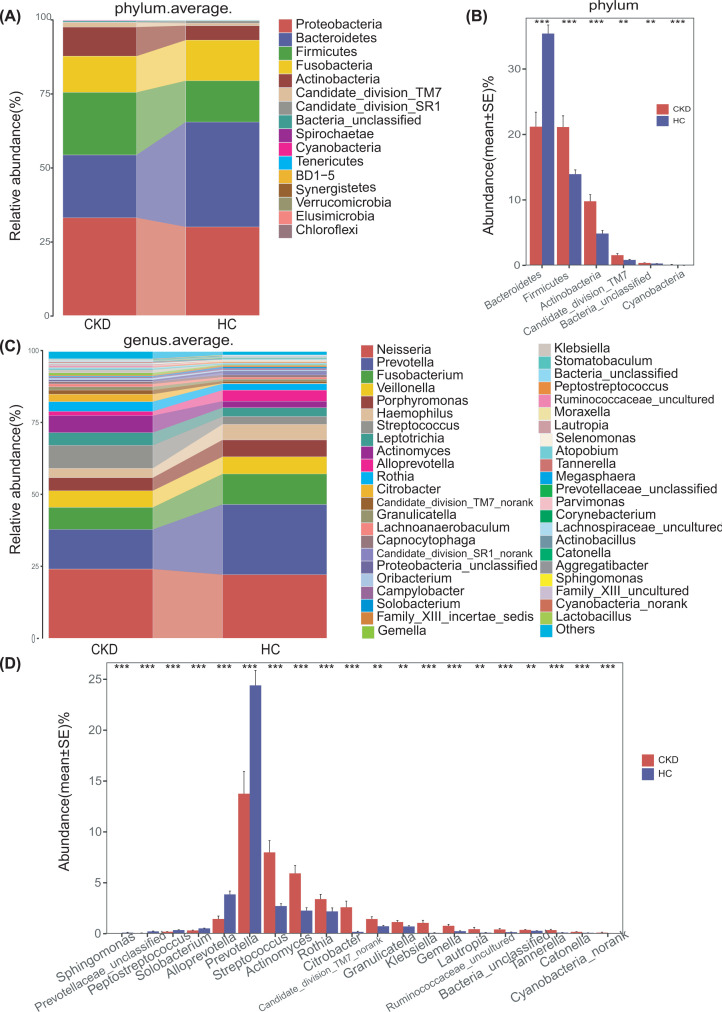
Differences in species levels of CKD (*n*=44) and HCs (*n*=88) oral microorganisms (**A,C**) The barplots show the comparison of the microbiome's relative abundance at the level of phylum (**A**) and genus (**C**) between CKD and HCs, respectively. The upper right corner is marked with a kind of bacteria represented by different colors, which is consistent with the main picture. (**B**) Five phyla were significantly enriched, while 1 phylum were significantly reduced in CKD (*n*=44) versus HCs (*n*=88). (**D**) Fourteen genera were significantly enriched, while 6 genera were significantly reduced in CKD (*n*=44) versus HCs (*n*=88). Wilcoxon test was used to compare significant differences between groups and FDR was calculated [Supplementary Data S5-6 (phylum) and S7-8 (Genus)]; Significant correlations by *Q<0.05; **Q<0.01 and ***Q<0.001(red, CKD; blue, HCs); CKD, chronic kidney disease; HC, healthy control.

The results of heatmap analysis (a hierarchical clustering analysis) of the oral microbiomes using a random forests model revealed a discriminatory oral microbiome between CKD and HCs groups. We delineated 57 key OTUs in the oral microbiome of the CKD and HCs groups shown in Figure 3 (Supplementary Data S4). In the CKD group, the oral microbiome was enriched in 34 OTUs, while the HCs group was enriched in 23 OTUs. Bacterial taxonomic composition between the CKD and HCs groups revealed 16 phyla, of which the four predominant phyla (*Proteobacteria, Bacteroidetes, Firmicutes* and *Fusobacteria*) accounted for 87% and 92% of the reads of the CKD and HCs samples, respectively (Figure 4A and Supplementary Data S5). Compared with the HC group, the abundance of *Bacteroides* in the CKD group was significantly decreased (*Q*<0.001). In contrast, abundance of *Firmicutes, Actinobacteria* and other 3 phyla were enriched in the CKD group (all *Q*<0.05) (Figure 4B and Supplementary Data S6). At the genus level, *Neisseria, Prevotella* and *Fusobacterium* are the three dominant populations in the two groups (Figure 4C and Supplementary Data S7). *Streptococcus, Actinomyces* and other 12 genera were more abundant in the CKD group (*Q*<0.05). However, the abundance of *Prevotella, Alloprevotella* and other 4 genera in the HCs was notably higher than in the CKD (*P*<0.05) (Figure 4D and Supplementary Data S8).

Furthermore, we compared the oral microbial composition between CKD and HCs at the class, order and family levels. The barplot shows the abundance and composition of flora with different biological grades (phylum, Supplementary Figure S3; genus, Supplementary Figure S4; class, Supplementary Figure S5; order Supplementary Figure S8; family, Supplementary Figure S11, respectively). And the average composition and relative abundance of the bacterial community in both groups at the three levels were shown in the Supplementary Figures S6, S9 and S12, respectively (Supplementary Data S16, S18, S20). Analysis at the class level showed that the abundance of the five bacterial classes including *Bacilli* and *Actinobacteria* increased significantly in the CKD group. And the abundances of *Alphaproteobacteria, Erysipelotrichia* and *Bacteroidia* are significantly increased in the HC group (*Q*<0.05) (Supplementary Figure S7, Data S17). At the order level, the abundance of 9 bacterial targets including *Lactobacillales, Actinomycetales* and *Enterobacteriales* increased significantly in the CKD group, while the abundance of *Erysipelotrichales, Pasturellales, Sphingomonadales* and *Bacteroidales* increased significantly in the HC group (*Q*<0.05) (Supplementary Figure S10, Data S19). At the bacterial family level, the abundance of 13 bacterial families including *Enterobacteriaceae, Actinomycetales* and *Streptococcaceae* was significantly increased in the CKD group, while the abundance of *Sphingomonadaceae, Erysipelotrichaceae, Pasturellaceae* and *Prevotellaceae* was significantly increased in the HCs group (*Q*<0.05) (Supplementary Figure S13, Data S21).

### LEfSe reveals significant microbial dysbiosis between the CKD and HCs oral samples

**Figure 5:**
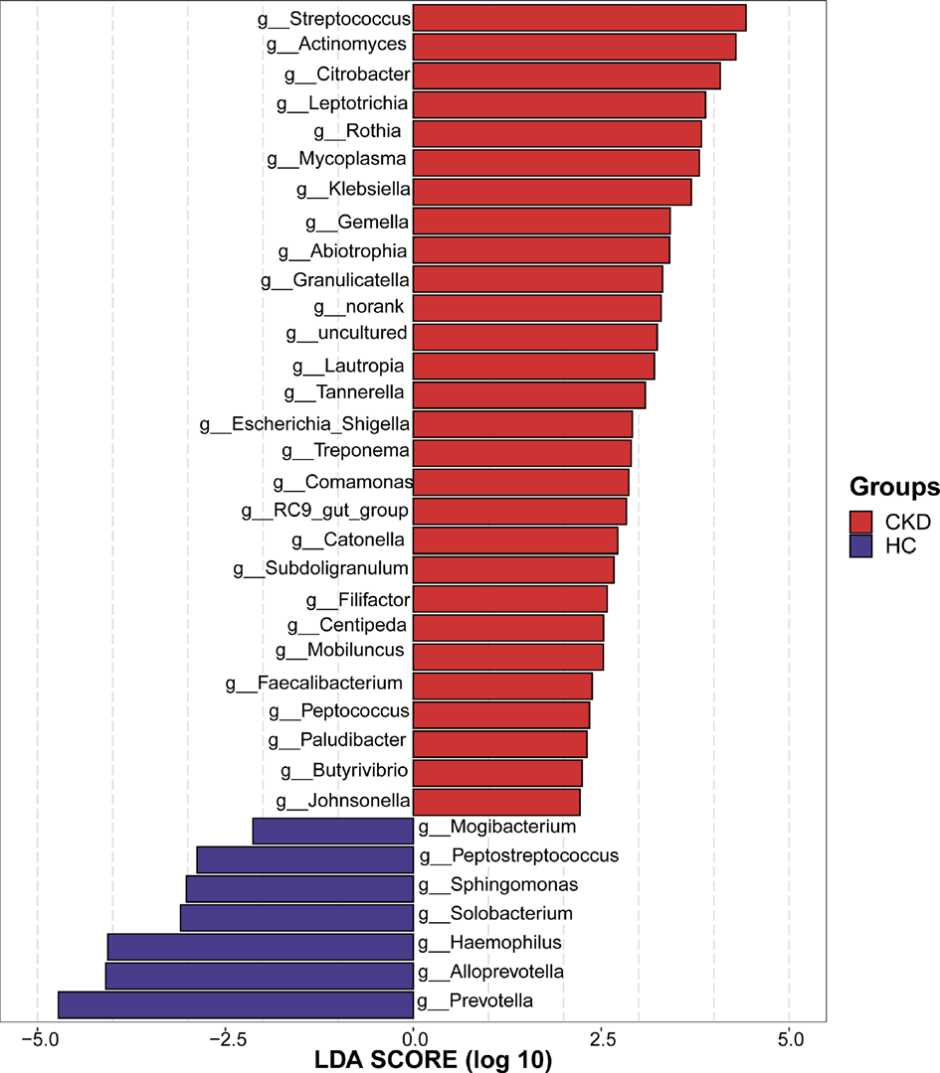
Crucial bacteria of oral microbiome related to CKD A histogram of the linear discriminant analysis (LDA) scores was calculated for the selected taxa which showed the significant bacterial difference between the CKD (*n*=44) and HCs (*n*=88) at the genus level. LDA score at the log10 scale is indicated at the bottom. The greater the LDA score is, the more significant the microbial biomarker is in the comparison. Based on the LDA selection, 28 genera were significantly enriched, while 7 genera were significantly reduced in CKD (*n*=44) compared with HCs (*n*=88) (all *P*<0.05) (Supplementary Data S9); CKD, chronic kidney disease; HC, healthy control; LDA, linear discriminant analysis.

The microbes of the oral of CKD patients was dominated in 28 bacterial genera including *Streptococcus, Actinomyces and Citrobacter* [LDA score (log10) > 2], whereas 7 bacterial genera including *Prevotella, Alloprevotalla and Haemophilus* are the predominant flora in the HCs oral [LDA score (log10) > 2) (Figure 5 and Supplementary Data S9).

**Figure 6:**
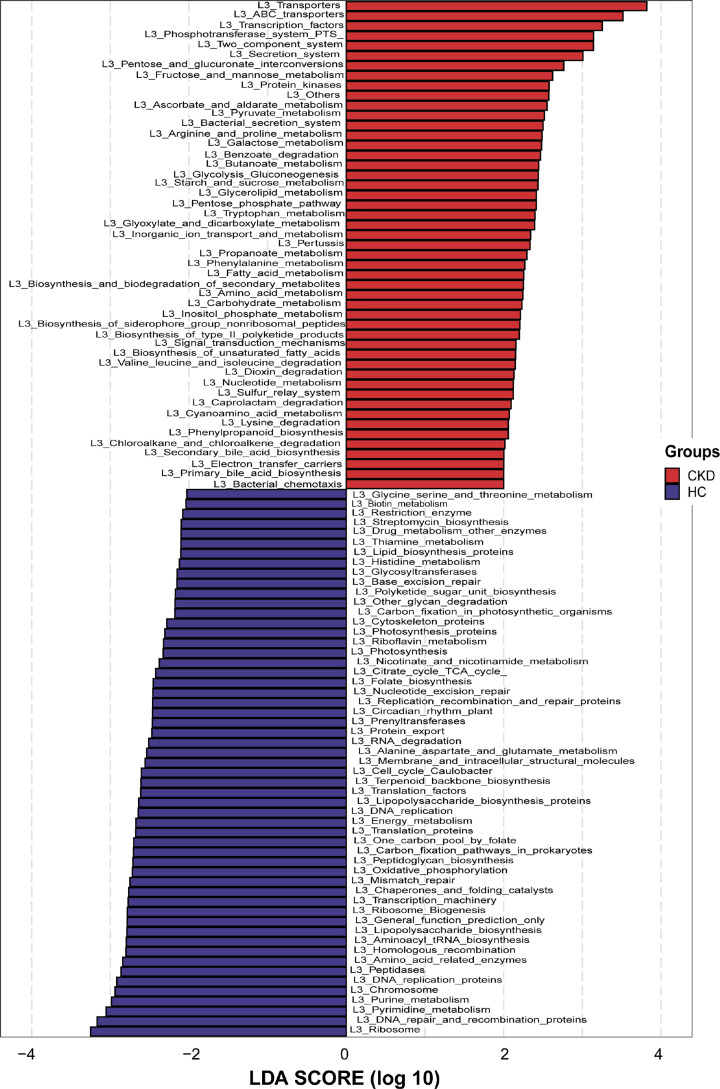
LDA scores predict gene functions associated with oral microbiomes in CKD (*n*=44) and HCs (*n*=88) using PICRUSt LDA score at the log10 scale is indicated at the bottom. The greater the LDA score is, the more significant the microbial biomarker is in the comparison. Based on the LDA selection, 49 predicted microbial functions were significantly increased,while 55 functions were remarkably decreased in CKD (*n*=44) compared with HCs (*n*=88) (all *P*<0.05) (Supplementary Data S10); CKD, chronic kidney disease; HC, healthy control; LDA, linear discriminant analysis.

We predicted the microbial community function based on 16S rRNA metagenomic sequencing results. Based on LDA selection, 49 microbial prediction functions including transporters, transcription factors, phosphotransferase system dominated the CKD group. However, 55 microbial prediction functions including ribosome, DNA repair and recombination proteins and pyrimidine metabolism dominate in HCs (Figure 6 and Supplementary Data S10). These differences indicated that oral microbiome and their related gene function changed in patients with CKD.

### Diagnostic potential of oral microbial markers of CKD

**Figure 7:**
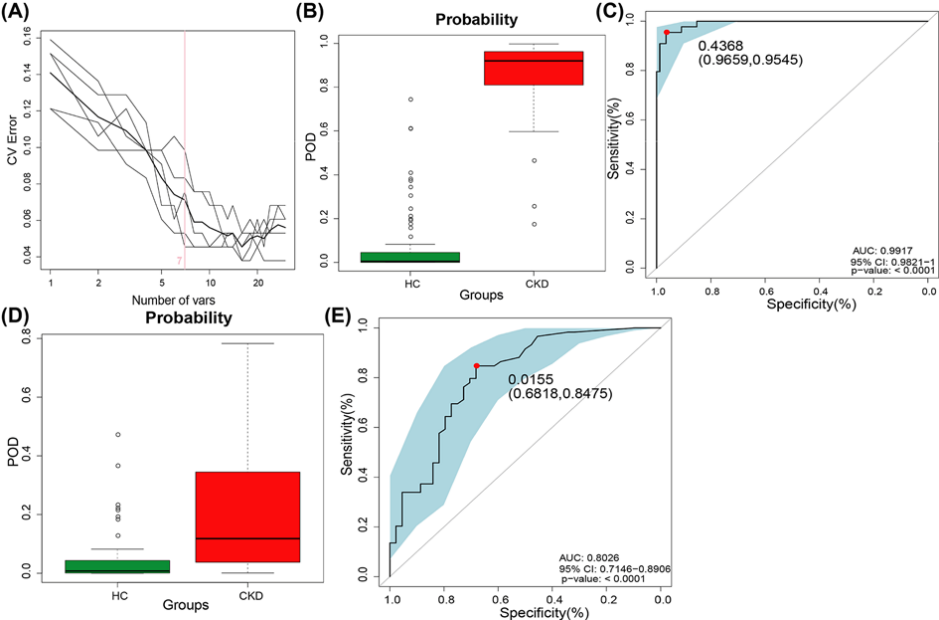
Diagnostic potential of oral microbiome markers in CKD patients (**A**) The 7 OTUs markers were selected as the optimal marker set by random forest models. (**B**) The POD value was remarkably increased in CKD (*n*=44) versus HCs (*n*=88) in the discovery phase. (**C**) The POD index achieved an AUC value of 0.9917 with 95% CI of 0.9821 to 1 between CKD (*n*=44) versus HCs (*n*=88) in the discovery phase (*P*<0.001). (**D**) The POD values were increased in CKD (*n*=59) compared with HC (*n*=44) (*P*<0.001). (**E**) The POD index achieved an AUC value of 0.8475 with 95% CI of 0.7146 to 0.8906 between CKD (*n*=59) versus HCs (*n*=44) in the validation phase (*P*<0.001) (Supplementary Data S11–S14); AUC, area under the curve; CI, confidence interval; CKD, chronic kidney disease; HC, healthy control; POD, probability of disease; ROC, receiver operating characteristic.

The random forest model was used to identify OTUs with diagnostic potential, and five-fold cross-validation was performed on the random forest model to further calculate POD. In the discovery phase, we constructed a random classifier model between 44 CKD patients and 88 HCs to prove the diagnostic value of oral microbiome for CKD. Seven OTUs markers were selected as the optimal marker set (Figure 7A and Supplementary Data S11), including OTU199 (*Citrobacter*), OTU1471 (*Actinomyces*) and OTU548 (*Prevotella*). The microflora data and the seven OTU biomarkers were used to calculate the POD index. The POD value of CKD was significantly higher compared with HCs (*P*<0.05, Figure 7B, Supplementary Data S12). And the POD index reached an area under the receiver operating characteristic (ROC) curve (AUC) of 0.9917 (95% CI: 0.9821–1, *P*<0.001) between CKD and HCs. These outcomes suggested the POD based on microbial markers achieved a powerful diagnostic potential for CKD cohort from the HCs cohort.

To further validate the diagnostic potential of markers, 58 CKD samples from Zhejiang (southeast China) were collected and completed Miseq Sequencing. The results showed that the POD value of the CKD group was significantly higher versus HCs group (*P*<0.05) (Figure 7D). And the POD index reached an AUC value of 0.8026 (95% CI: 0.7146–0.8906, *P*<0.001) (Figure 7E and Supplementary Data S13–14). These results achieve cross-region validation, and the CKD-associated microbial genera distinguish CKD from HCs.

### Correlation between the oral microbiome and clinical indicators of CKD

**Figure 8:**
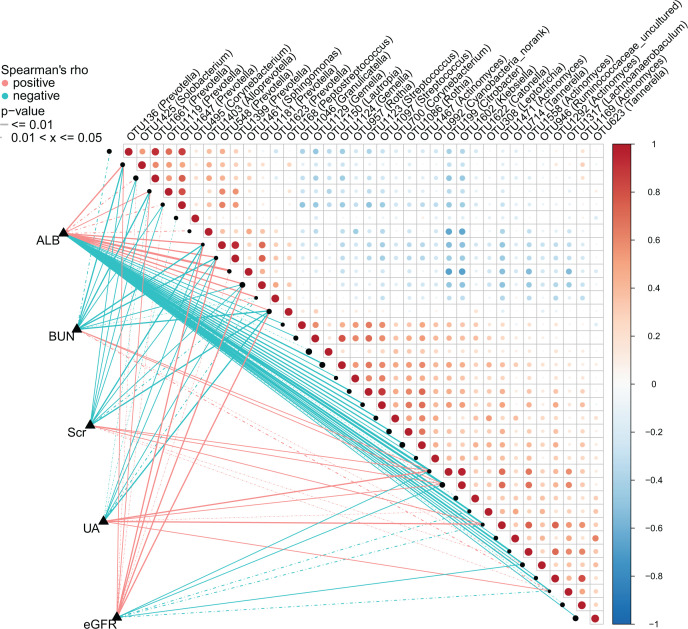
Heatmap showing the partial Spearman’s correlation coefficients among 36 OTUs and 5 clinical indicators of CKD (*n*=44) Distance correlation plots of relative abundances of 36 OTUs and the clinical indices (ALB, BUN, SCr, UA, eGFR). *Q* values are shown in Supplementary Data S15; ALB, albumin; BUN, blood urea nitrogen; CKD, chronic kidney disease; eGFR, estimated glomerular filtration rate; OTU, operational Taxonomy Unit; SCr, serum creatinine; UA, uric acid.

Based on the Spearman correlation analysis, we further analyzed the correlations between the oral microbiome and clinical indicators of CKD (*n*=44), including WBC, RBC, GLOB, ALB, BUN and other 11 indicators, and found 5 clinical indicators (ALB; BUN; Scr; UA; eGFR) were closely related to the 36 OTUs of CKD (Figure 8 and Supplementary Data S15). ALB was positively correlated with 11 OTUs including OTU1623 (*Prevotella*) and OTU1181 (*Prevotella*), while negatively correlated with 23 OTUs including OUT 1129 (*Gemella*) and OTU 1150 (*Lautropia*) (*Q*<0.05). BUN was positively correlated with OTU 1371 (*Lachnoanaerobaculum*), OTU 1602 (*Klebsiella*), OTU 946 (*Ruminococcaceae_uncuitured*) and OTU 199 (*Citrobacter*), while negatively correlated with 10 OTUs including OTU1623 (*Prevotella*) and OTU1181 (*Prevotella*) (*Q*<0.05). Scr was positively correlated with 5 OTUs including OTU 946(*Ruminococcaceae_uncuitured*) and 1471(*Actinomyces*), while negatively correlated with 10 OTUs including OTU1623 (*Prevotella*) and OTU1181 (*Prevotella*) (*Q*<0.05). The correlation between other two indicators such as UA and eGFR and oral microbes is shown in Figure 8.

## Discussion

Studies have shown that disorders of human oral microbiota are associated with multiple diseases. *Leptotrichia, Fusobacterium* other 11 bacteria were overrepresented in the oral of pancreatic head carcinoma (PHC) patients [12]. The liver carcinoma (LC) microbiome was characterized by a preponderance of 5 bacteria, including *Fusobacteria, Clostridia* and *Actinobacteria* [13]. Casarin et al. found higher percentages of 6 bacteria, including *Fusobacterium* and *Streptococcus* genera in the mouths of patients with type 2 diabetes [18]. Koren et al. found that *Staphylococcus aureus, Veillonella* and *Streptococcus* are found in the oral microbiome of most patients with atherosclerosis [19]. Kshirsagar et al. studied the correlation between serum antibodies of oral pathogenic microorganisms and renal function, and found that periodontal pathogens including *Porphyromonas gingivalis, Treponema pallidum* and *Haemophilus actinomycete* were associated with serum IgG levels and renal function, and negatively correlated with eGFR [20]. However, the characteristics of oral microbiome communities in CKD patients are rarely reported. This study is the first time to describe the characteristic of the CKD oral microbiome, and is the first time to conduct the CKD noninvasive diagnostic model based on biomarkers.

The oral microbiome has received extensive attention, which can be used as potential biological markers for disease diagnosis and has unique advantages. This study finally sequenced 235 oral samples from different parts of China, elucidate the characteristics of the oral microbiome in CKD patients, using the random forest model to identify 7 optimal microbial markers, and conducted the CKD classifier. Furthermore, the validation set from Hangzhou (southeast, China) was used to verify the results and indicated that have higher diagnostic potential. Studies have shown that the influence of environmental factors on oral microbes is significantly greater than genetic factors [21]. Lifestyle, geographical environment and acute infections are the main influencing factors of oral microbiome [22], and there are significant individual differences. Notably, our research is the first to achieve cross-regional invalidation of the CKD classifier model based on oral microbial markers, which reduces the impact of variability to a certain extent.

Disease inters by the mouth. Adults produce a lot of saliva every day, almost all enter the gastrointestinal tract. Oral microbiome enters the gastrointestinal tract along with saliva or food, and the population repetition rate between oral microbiome and intestinal flora can reach 45% [23]. A high abundance of oral microbiome has been detected in the intestines of diseases such as acute appendicitis [24], inflammatory bowel disease (IBD), colorectal cancer (CRC) and other tissues [25]. Schmidt et al. found that 59% of oral microbes frequently migrate and colonize the intestinal tract, and the multiple colonies enriched in the intestines of patients with RA and CRC are all derived from the oral cavity [26]. Microorganisms present in the oral cavity were found in the intestinal examination of CKD patients, such as *Firmicutes, Actinobacteria* and *Alloprevotalla* [27]. We previously studied the characteristics of the intestinal flora of nondialysis CKD patients [28] and found that Actinomycetes are not only enriched in the intestine but also dominant in oral microbiome. It suggests that the oral microbiome may be involved in the occurrence and development of some diseases to a certain extent.

Our results suggest that the oral microbiome has changed significantly in CKD patients. Compared with the HC group, we found the bacterial diversity of CKD patients increased. Five phyla were more abundant in the CKD group, among which *Actinomycetes* can trigger neutrophils to release matrix metalloproteinases (MMP), and stimulate macrophage-like cells to secrete more MMP *in vitro* [29,30]. MMP elevation is considered to be a potential marker of tissue destruction in inflammation [31]. A total of 14 genera were more abundant in the CKD group. Streptococcus may cause the chemotaxis of Th22 cells to kidney tissue, leading to or aggravating kidney injury [32], which negatively correlated with renal function [33]. We further detected seven optimal OTU markers set between discovery phase, including OTU199 (*Citrobacter*), OTU1471 (*Actinomyces*), and OTU1602 (*Klebsiella*), indicating that these key florae may be distinguished CKD from HC, which has been further verified and has a high diagnostic potential. Among them, and *Klebsiella* can produce lipopolysaccharide, which is an immune active component and participates in the host’s innate immune process. It can activate the nuclear transcription factor NF-κB classic inflammation signaling pathway, causing the inflammatory factor interleukin (IL)-1β, IL-6, IL-8, TNF-α increased expression, leading to inflammation [34]. Evidence suggests that inflammation and metabolic disorders may be involved in changes in the oral microbiome of CKD patients, resulting in increased salivary urea concentration. Patients with CKD experience changes in salivary pH and flow rate, which exacerbate oral diseases [35]. The alkaline oral environment is more conducive to pathogenic bacteria and colonization in the oral cavity [36]. Ammonia involvement is known to cause mucosal inflammation, increase the chance of infection and may increase kidney damage [37,38]. Most importantly, the low level of inflammation is one of the important risk factors for CKD procession [39]. In addition, the study of the gene function of the microbiome found significant differences in the CKD group. Compared with HC, forty-nine dominant microbial functions were identified in the CKD group, which confirmed that the CKD oral microbiome had changed. Arginine metabolism can produce asymmetric dimethylarginine (AMDA) and polyamines. AMDA, as a nitric oxide synthase inhibitor, causes endothelial dysfunction, vasoconstriction, oxidative stress and atherosclerosis by inhibiting the formation of nitric oxide. ADMA accumulation can also be used as cardiovascular disease in CKD patients [40]. Increased tryptophan metabolism leads to increased production of indoxyl sulfate, which is inversely related to eGFR [41]. Such studies may identify the mechanisms of interaction between the CKD and the oral microbiome. Therefore, changes in the oral microbiome of CKD patients suggest that they may have important physiological significance in the occurrence and development of CKD, and the targeted biomarkers based on oral microbes are expected to become potential non-invasive diagnostic tools for CKD, which is worthy of further exploration.

Furthermore, we analyze the correlations between the oral microbiome and clinical indicators of CKD. *Lachnoanaerobaculum* and *Ruminococcaceae_uncuitured* are positively correlated with BUN and Scr, and negatively correlated with eGFR. In addition, *Peptostreptococcus* and *Prevotella* are negatively correlated with BUN and Scr, and positively correlated with eGFR. The results show that it is of great significance to provide a new idea for the diagnosis of CKD and a new direction for the treatment of CKD based on the oral microbiome.

In summary, we clarified the characteristics of the oral microbiome of CKD patients, identified disease-related microbial markers and demonstrated the potential as a noninvasive diagnostic tool for CKD. The identification of oral microbiome of different races or different countries may provide greater support for the diagnosis of CKD by oral microbes, making it more effective and universal. Moreover, the occurrence and development of CKD are closely related to metabolites, which is essential for metabolomics analysis. This is the shortcoming of this research and the focus of current work. Therefore, these findings confirm the malnutrition of the oral microbiome of CKD, which can be used to develop new methods for noninvasive diagnosis and intervention and treatment of CKD.

## Supplementary Material

Supplementary Figures S1-S14

Supplementary Data S1-S21

## Data Availability

The raw Illumina read data for all samples were deposited in the European Bioinformatics Institute European Nucleotide Archive database under the accession number PRJNA557511(HC group) and PRJNA649074(CKD group).

## Abbreviations

ALB: albumin
BMI: body mass index
BUN: blood urea nitrogen
CKD: chronic kidney disease
eGFR: estimated glomerular filtration rate
ESRD: end-stage renal disease
HC: health control
LDA: linear disciminant analysis
LEfSe: linear disciminant analysis effect size
NMDS: nonmetric multidimensional scaling
OTU: operational taxonomic unit
PCA: principal component analysis
PCoA: principle co-ordinates analysis
PCOS: polycystic ovary syndrome
PCR: polymerase chain reaction
PICRUSt: Phylogenetic Investigation of Communities by Reconstruction of Unobserved States
RNA: ribonucleic acid
ROC: receiver operating characteristic
SCr: serum creatinine
UA: uric acid
