# Supplementary material for: Characteristics of human oral microbiome and its non-invasive diagnostic value in chronic kidney disease
Source: Biosci Rep. 2022 May 10;42(5):BSR20210694. doi: 10.1042/BSR20210694 (PMC9093701; doi:10.1042/BSR20210694)
Supplement: Supplementary Figures S1-S14 [file BSR-2021-0694_supp.pdf]

Supplementary figures legends

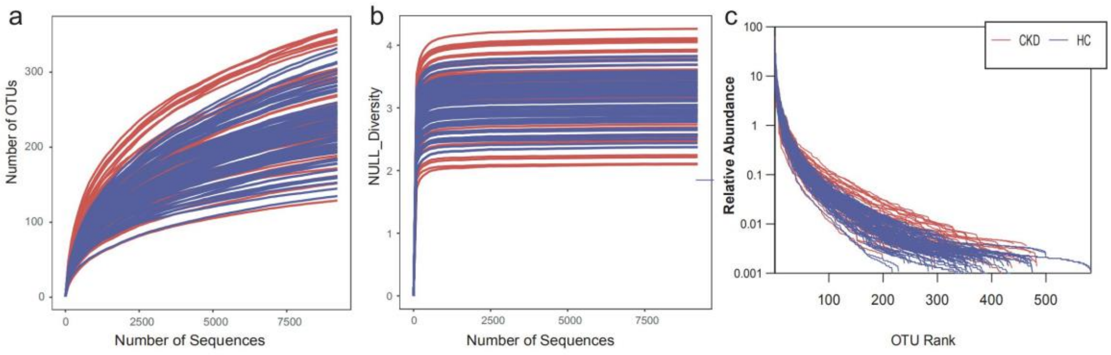

Figure S1. Alpha diversity in the oral of CKD patients. (a) A rarefaction curve between the number of OTUs and the number of sequences in CKD (n=44) and HCs (n=88); (b) A shannon-wiener curve between the number of sequences and the null diversity in CKD (n=44) and HCs (n=88); (c) A rank-abundance distribution curve for the OTUs of CKD (n=44) and HCs (n=88). CKD, chronic kidney disease; HCs, healthy controls; OTUs, operational taxonomic units.

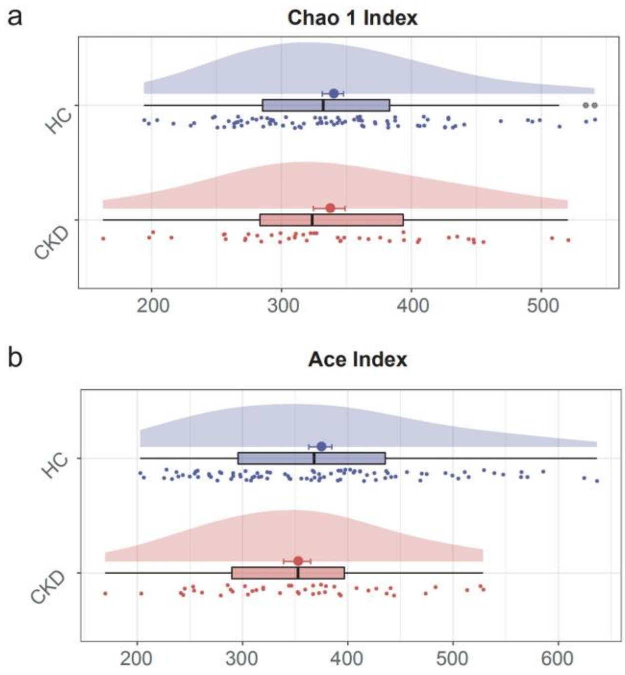

Figure S2. Chao 1 and Ace indices for CKD (n=44) and HCs (n=88). (a) Estimated by Chao1 index, community richness no significant difference between CKD and HCs. (b) Ace index indicates that oral microbial abundance has no significant difference between CKD and HCs. CKD, chronic kidney disease; HCs, healthy controls.

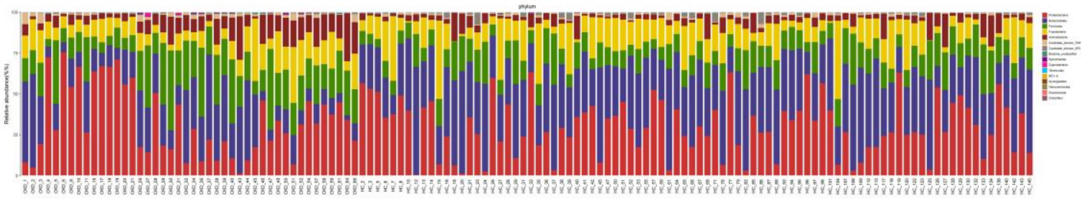

Figure S3. The composition and abundance of bacterial community at the phylum level in each sample of the discovery group (n=132). CKD, chronic kidney disease; HCs, healthy controls.

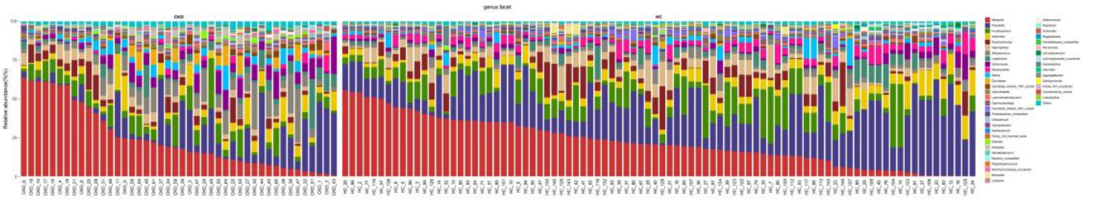

Figure S4. The composition and abundance of bacterial community at the genus level in each sample of the discovery group (n=132). CKD, chronic kidney disease; HCs, healthy controls.

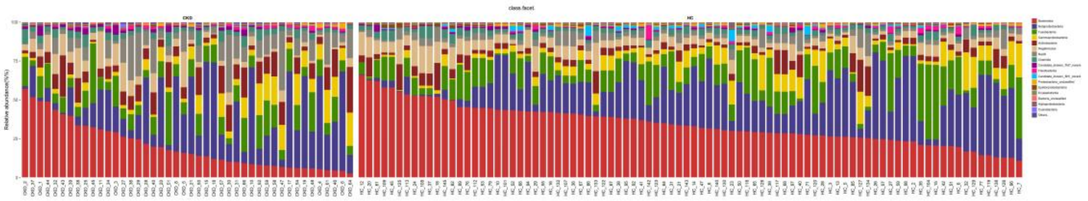

Figure S5. The composition and abundance of bacterial community at the class level in each sample of the discovery group (n=132). CKD, chronic kidney disease; HCs, healthy controls.

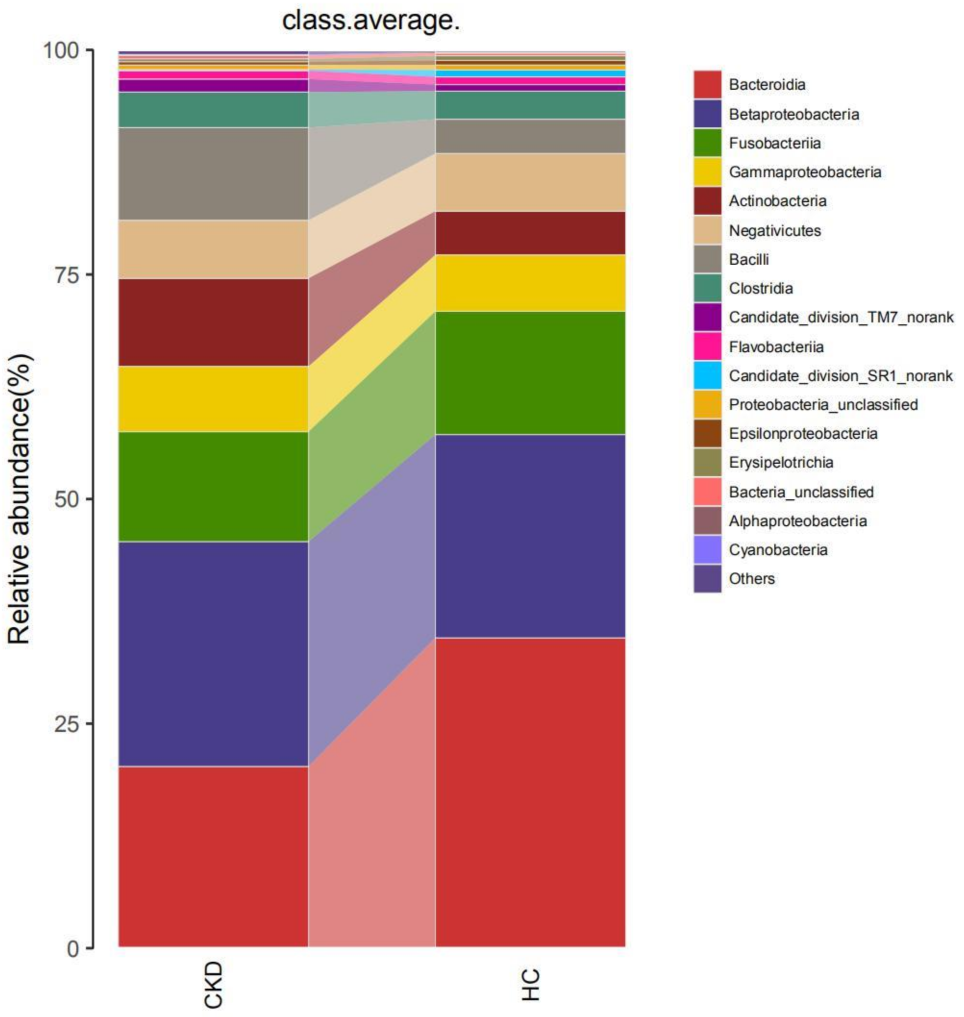

Figure S6. The average composition and relative abundance of the bacterial communities of CKD (n=44) and HCs (n=88) at the class level in the discovery group. CKD, chronic kidney disease; HCs, healthy controls.

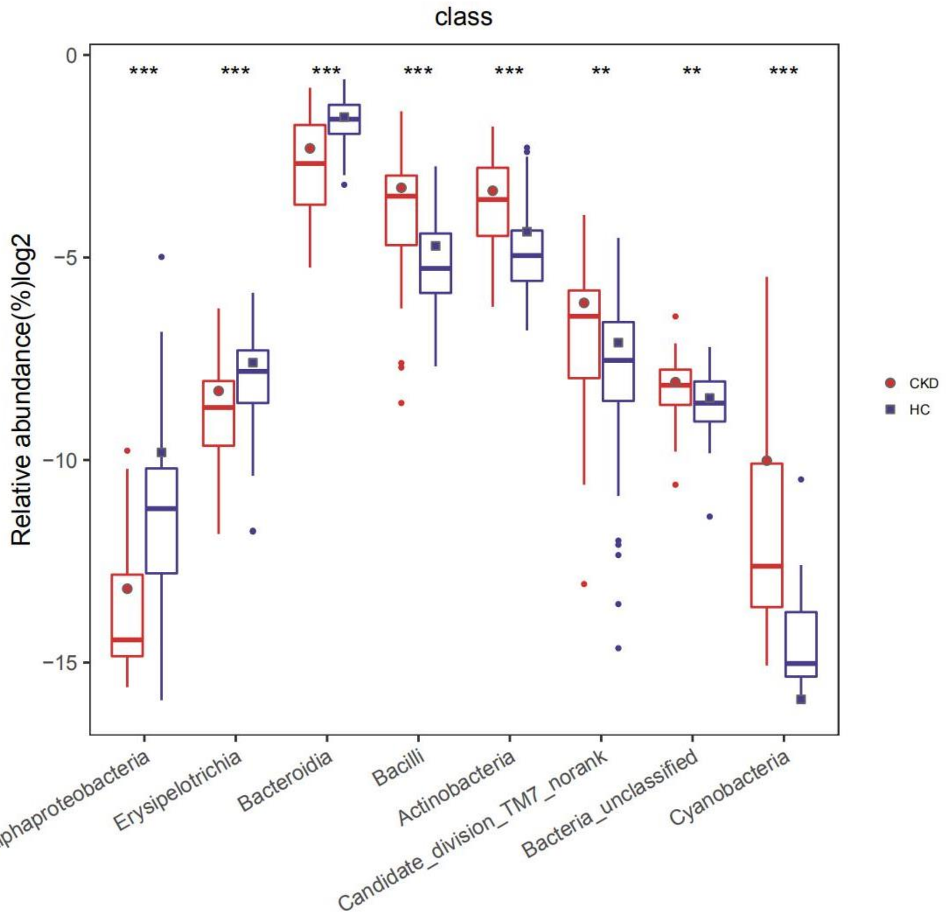

Figure S7. At the class level, 5 bacterial populations were significantly enriched, whereas 3 bacterial populations were significantly reduced in CKD (n=44) versus HCs (n=88). **, Q<0.01, ***, Q<0.001. CKD, chronic kidney disease; HCs, healthy controls.

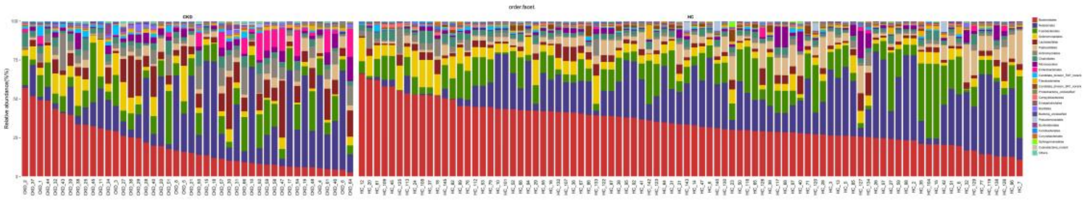

Figure S8. The composition and abundance of bacterial community at the order level in each sample of the discovery group (n=132). CKD, chronic kidney disease; HCs, healthy controls.

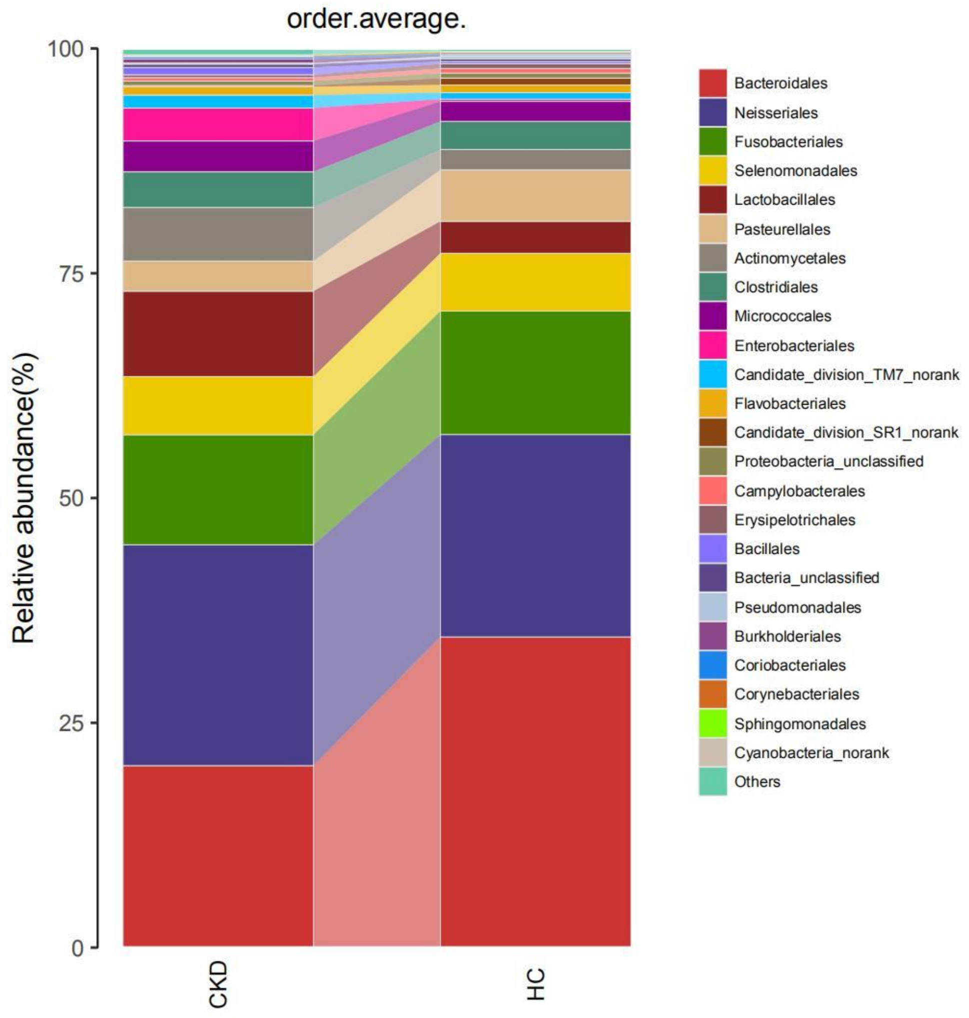

Figure S9. The average composition and relative abundance of the bacterial communities of CKD (n=44) and HCs (n=88) at the order level in the discovery group. CKD, chronic kidney disease; HCs, healthy controls.

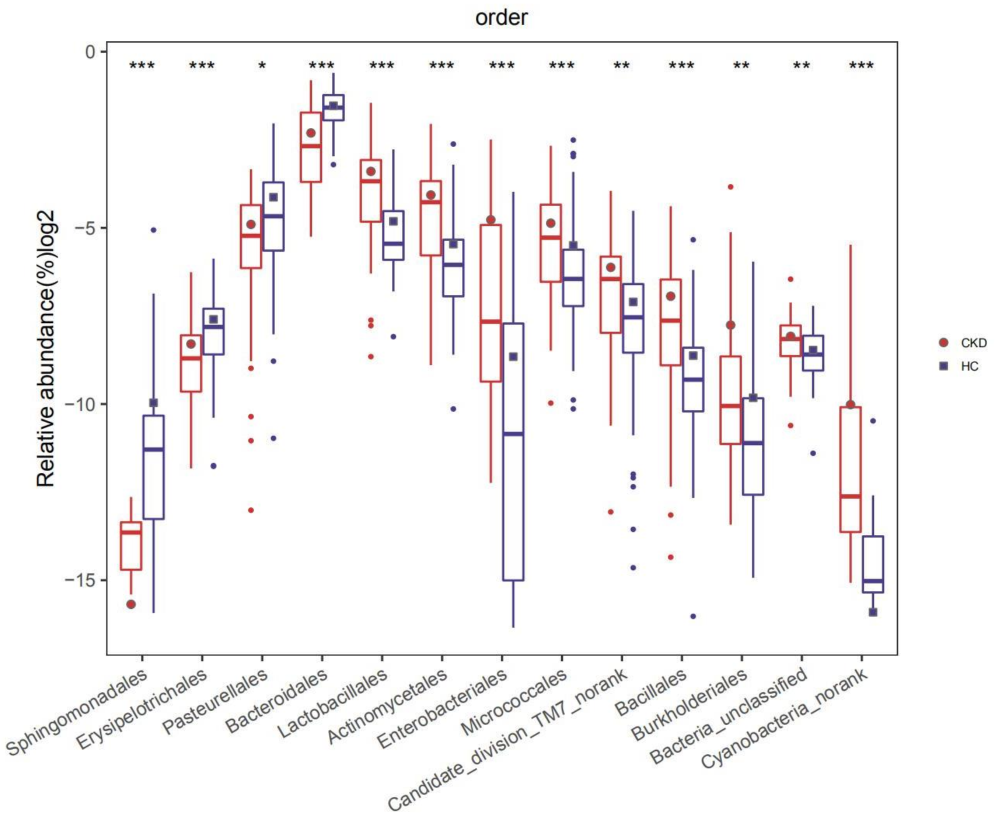

Figure S10. At the order level, 9 bacterial populations were significantly enriched, whereas 4 bacterial populations were significantly reduced in CKD (n=44) versus HCs (n=88). *, Q<0.05, **, Q<0.01, ***, Q<0.001. CKD, chronic kidney disease; HCs, healthy controls.

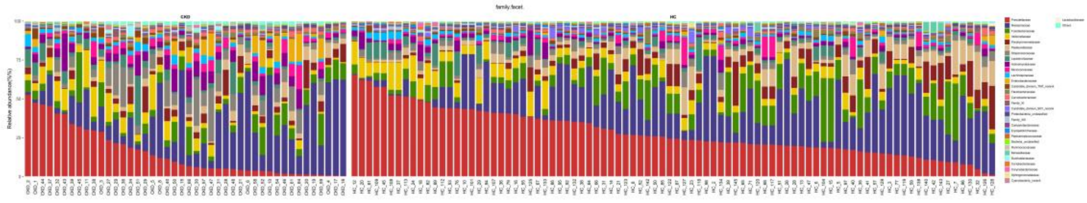

Figure S11. The composition and abundance of bacterial community at the family level in each sample of the discovery group (n=132). CKD, chronic kidney disease; HCs, healthy controls.

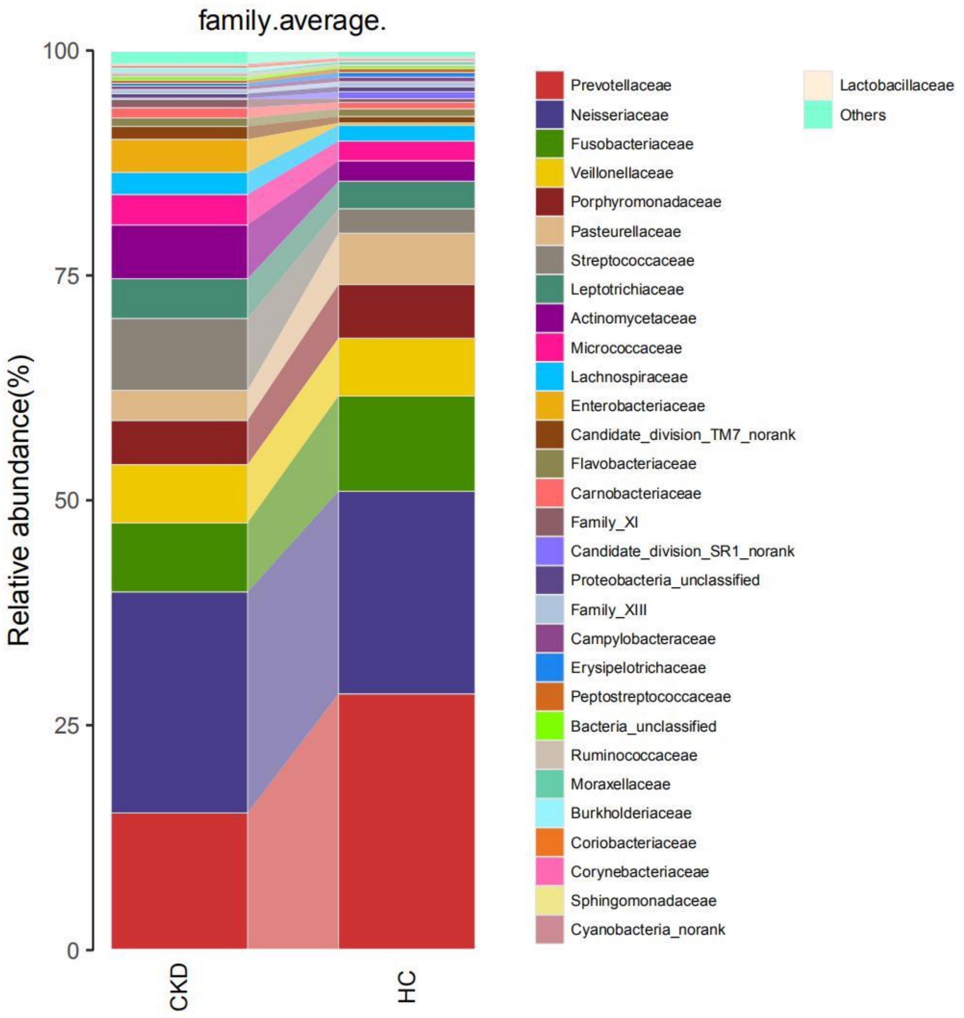

Figure S12. The average composition and relative abundance of the bacterial communities of CKD (n=44) and HCs (n=88) at the family level in the discovery group. CKD, chronic kidney disease; HCs, healthy controls.

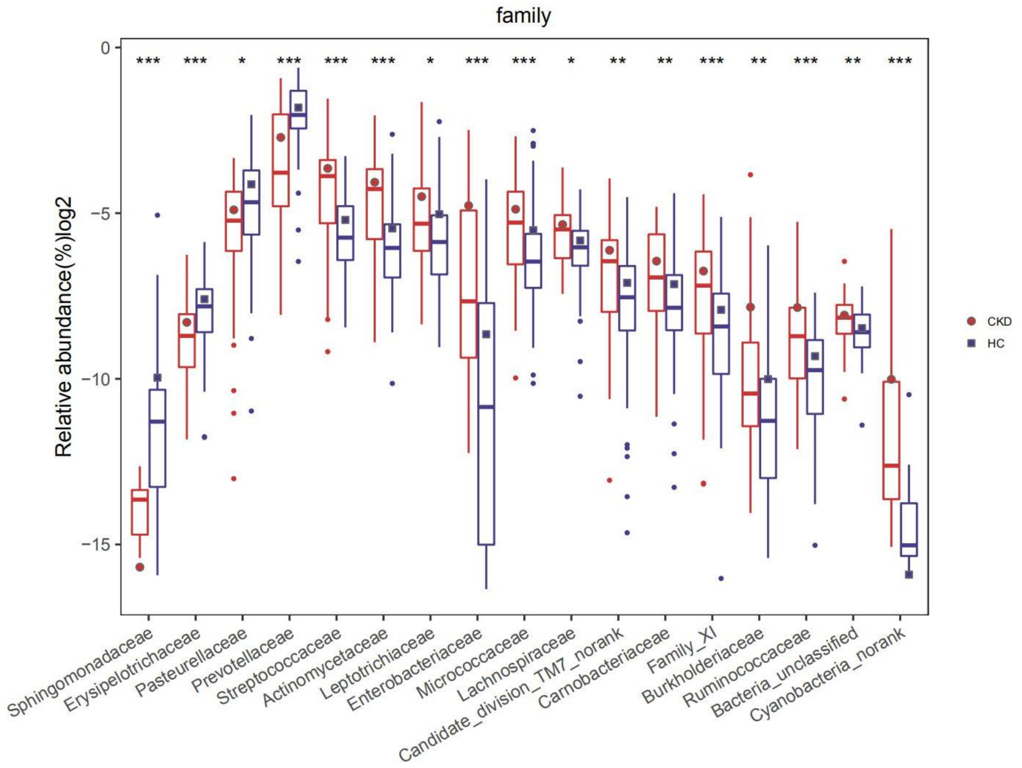

Figure S13. At the family level, 13 bacterial populations were significantly enriched, whereas 4 bacterial populations were significantly reduced in CKD (n=44) versus HCs (n=88). *, $Q<0.05$, **, $Q<0.01$, ***, $Q<0.001$. CKD, chronic kidney disease; HCs, healthy controls.

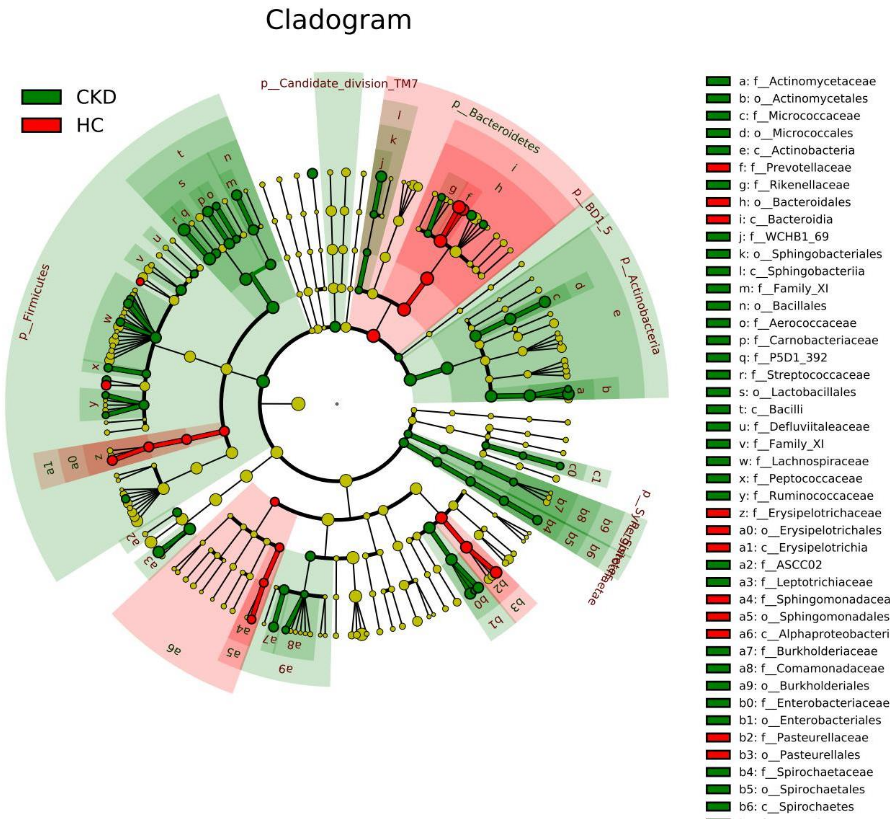

Figure S14. Cladogram using the LEfSe method indicating the phylogenetic distribution of oral microbes associated with patients with CKD(n=44) (green indicates phylotypes statistically overrepresented in CKD) and HCs (n=88)(red indicates phylotypes overrepresented in healthy controls). Each filled circle represents one phylotype, and phylum and class are indicated in their names on the cladogram and the order, family, or genera are given on the right panel. CKD, chronic kidney disease patients; HCs, healthy controls.
