# Supplementary material for: Characteristics of human oral microbiome and its non-invasive diagnostic value in chronic kidney disease
Source: Biosci Rep. 2022 May 10;42(5):BSR20210694. doi: 10.1042/BSR20210694 (PMC9093701; doi:10.1042/BSR20210694)
Supplement: Supplementary Data S1-S21 [file BSR-2021-0694_supp1.zip › BSR-2021-0694_suppD.pdf]

Data S1. Clinical data of all Chinese individuals in the discovery group (n=132), validation group (n=103).

Data S2. The detailed values of oral microbiome diversity index and observed OTUs in the discovery group (88 HCs and 44 CKD).

Data S3. The corresponding PCoA value for each sample in discovery group (88 HCs sand 44 CKD).

Data S4. The relative abundance and distribution of the key 57 OTUs in the discovery group (88 HCs and 44 CKD).

Data S5. The abundance and composition at the phylum level of each sample in the discovery group (88 HCs and 44 CKD).

Data S6. The different degree of phylum level (q value) between the HCs (n=88) and CKD (n=44) in the discovery group.

Data S7. The abundance and composition at the genus level of each sample in the discovery group (88 HCs and 44 CKD).

Data S8. The different degree of genus level (q value) between the HCs (n=88) and CKD (n=44) in the discovery group.

Data S9. The corresponding LDA value and p value of the bio-makers in the discovery group (88 HCs and 44 CKD).

Data S10. The corresponding LDA value and p value of microbial community gene function for samples in the discovery group (88 HCs and 44 CKD).

Data S11. By random forest classifier model, the corresponding output value of each optimal microbial marker in the discovery group (88 HCs and 44 CKD).

Data S12. The corresponding POD value for each sample in discovery group (88 HCs and 44 CKD).

Data S13. By random forest classifier model, the corresponding output value of each optimal microbial marker in the validation group (59 CKD from Hangzhou, china and 44 HCs).

Data S14. The corresponding POD value for each sample in validation group (44 HCs and 59 CKD).

Data S15. The data of canonical correspondence analysis (CCA) between oral microbiome and clinical indicators of CKD.

Data S16. The abundance and composition at the class level of each sample in the discovery group (88 HCs and 44 CKD).

Data S17. The different degree of class level (q value) between the HCs (n=88) and CKD (n=44) in the discovery group.

Data S18. The abundance and composition at the order level of each sample in the discovery group (88 HCs and 44 CKD).

Data S19. The different degree of order level (q value) between the HCs (n=88) and CKD (n=44) in the discovery group.

Data S20. The abundance and composition at the family level of each sample in the discovery group (88 HCs and 44 CKD).

Data S21. The different degree of family level (q value) between the HCs (n=88) and CKD (n=44) in the discovery group.
